# *Lacticaseibacillus paracasei* as a Modulator of Fatty Acid Compositions and Vitamin D3 in Cream

**DOI:** 10.3390/foods11111659

**Published:** 2022-06-05

**Authors:** Michał Złoch, Katarzyna Rafińska, Mateusz Sugajski, Magdalena Buszewska-Forajta, Justyna Walczak-Skierska, Viorica Railean, Paweł Pomastowski, Dorota Białczak, Bogusław Buszewski

**Affiliations:** 1 Department of Environmental Chemistry and Bioanalytics, Faculty of Chemistry, Nicolaus Copernicus University in Toruń, Gagarina 7 St., 87-100 Torun, Poland; michalzloch87@gmail.com (M.Z.); mateusz.sugajski@o2.pl (M.S.); walczak-skierska@umk.pl (J.W.-S.); bbusz@umk.pl (B.B.); 2 Centre for Modern Interdisciplinary Technologies, Nicolaus Copernicus University in Toruń, Wileńska 4 St., 87-100 Torun, Poland; vioricarai@umk.pl (V.R.); p.pomastowski@umk.pl (P.P.); 3 Institute of Veterinary Medicine, Faculty of Biological and Veterinary Sciences, Nicolaus Copernicus University in Toruń, Lwowska 1, 87-100 Toruń, Poland; m.buszewska@umk.pl; 4 POLMLEK Grudziądz Sp. z o. o., Magazynowa 8 St., 86-300 Grudziądz, Poland; d.bialczak@polmlek.com

**Keywords:** vitamin D3, Lactobacillus, cream, fatty acids

## Abstract

Butter is an important source of essential fatty acids, lipid-soluble vitamins, and antioxidants in the diet. However, this study showed that the presence of the *Lacticaseibacillus paracasei* strain has a great influence on the fatty acid profile as well as provitamin D3 and vitamin D3 content in the cream—the raw material from which the butter is obtained. The addition of this lactic acid bacteria enriches the cream in 9-hexadecenoic acid, oleic acid, octadeca-9,12-dienoic acid, and conjugated linoleic acid, which exhibit antimutagenic and anticarcinogenic properties. Moreover, a higher level of monounsaturated fatty acids can extend the shelf life of butter in the future. In the present work, we observed that the presence of lactic acid bacteria contributed to an increase in the level of provitamin D after 6 h of incubation and an increase in the levels of vitamin D3 after 24 and 48 h. Fatty acid profiles and the content of vitamins were largely dependent on the presence of light and mixing, which are probably associated with the status of lipid peroxidation.

## 1. Introduction

In recent years, there was a significant shift in consumer perceptions of food products. Considering the growing number of health-conscious consumers, functional foods that provide health benefits beyond basic nutrition have received particular attention [1]. Their functionalities may be associated with specific nutrients, such as vitamins, minerals, fiber, prebiotics, or probiotics, but they can deliver additional benefits above their basic nutritional values [2]. One key research area for the future functional food market is the development of probiotic food formulations, recognized as a major class of health-promoting foods that contain live microorganisms that, when administered in adequate amounts, confer health benefits on the host [3,4]. Due to the increasing demands for probiotic foods, lactic acid bacteria (LAB) are an industrially important group of microorganisms being used for the production of different kinds of fermented foods, since many may demonstrate desired probiotic properties [5].

In terms of economical and nutritional values, butter is considered an important dairy product since it contains essential fatty acids (FA) (e.g., linoleic acid, conjugated linoleic acid, eicosa-8,11,14-trienoic acid, and arachidonic acid) that cannot be synthesized in humans [6]. Due to the high content of fat, butter is also a good source of lipid-soluble vitamins, such as retinol, carotenoids, and tocopherols, which function as antioxidants that are important for human health [7]. Regarding vitamins, special attention is given to vitamin D, especially cholecalciferol (vit. D3), which plays a pivotal role in enhancing calcium and phosphorous absorption necessary for bone mineralization (calcemic functions) as well as in a variety of non-skeletal disorders (cancers, autoimmune diseases, cardiovascular disease, etc.) [8,9]. Since vitamin D deficiency has been recognized as a worldwide problem associated with many human disorders and very few foods contain vitamin D in sufficient amounts to obtain the desired health benefits, food fortification is a potentially effective strategy to increase vitamin D intake in humans [10]. In such terms, butter demonstrates high potential as a vitamin D carrier due to its high lipid content, including cholesterol, which is necessary for vitamin D3 synthesis.

Cream is the main raw material in the production of butter, the properties of which reflect the quality of the butter. Although the fermentation of cream with appropriate starter cultures of LAB is a well-known practice that implicates physicochemical and sensory properties of butter, and strongly influences its nutritional composition, textural properties, and shelf life, the use of LAB demonstrating probiotic properties in butter has been seldom reported in the literature [2]. Nevertheless, there are a few studies that have shown the abilities of probiotic bacteria to survive in butter at sufficient amounts to maintain probiotic effects, e.g., *Bifidobacterium bifidum* [7] and *Lactobacillus acidophilus* [6]. There is evidence that the utilization of probiotic bacteria in butter production can improve its health and nutritional properties by reducing the cholesterol content (on a fat basis) [11]. Moreover, many researchers report that bioactive fatty acids are formed by probiotic strains and can alter the fatty acid compositions of dairy products [12,13]. Ewe and Loo [14] observed that the addition of *Lactobacillus helveticus* during the cream maturation step resulted in better physicochemical and rheological properties of the butter (softer than the conventional) as a result of increased levels of unsaturated fatty acids (UFA). Caplice and Fitzgerald [15] noted that cultured butter is very often more aromatic and tangy than traditional non-fermented butter. Research has shown that probiotics demonstrate increased absorption of vitamin D and increased expression of the vitamin D receptor (VDR), and supplementation may increase serum levels of 25-hydroxyvitamin D3 in humans [16]. Additionally, probiotics are known to produce different kinds of metabolites, such as lactate, acetate, and pyruvate, which, when absorbed by human cells, lead to increased biosynthesis of 7-DHC (provitamin D3). Therefore, it is possible that when probiotic bacteria are added to butter, which also contains compounds contributing to the formation of vitamin D3, the biosynthesis of 7-DHC may increase and, consequently, the content of vitamin D3 in the dairy product will increase [17].

For this reason, the main goal of the study was to investigate the impact of the *Lacticaseibacillus paracasei* strain on the changes in the fatty acid composition, as well as cholecalciferol (vitamin D3) and its precursor level in the cream under different incubation conditions (i.e., light/dark and with/without mixing). We presumed that under certain conditions, the addition of LAB would demonstrate a beneficial effect on the cream characteristics—an increased unsaturated to saturated fatty acid ratio, higher provitamin, and/or vitamin D3 content—which could be further used for the preparation of a new probiotic butter formulation.

## 2. Materials and Methods

### 2.1. Chemicals

Sodium chloride, methanol, potassium carbonate, chloroform, and sulfuric acid (VI) were obtained from Sigma Aldrich (Steinheim, Germany), while hexane was purchased from Avantor (Gliwice, Poland). Deionized water was obtained using the system from Millipore (Schaffhausen, Switzerland). The cream was delivered by the Polmlek dairy (Grudziądz, Poland).

### 2.2. Obtaining Lacticaseibacillus paracasei Biomass

*L. paracasei* deposited at no. B/00287 at the Polish Collection of Microorganisms was cultured in MRS medium, a medium dedicated to the effective growing of LAB bacteria strains. Firstly, the bacteria stored in Cryobank™ tubes were cultured on the antibiograms loaded with De Man, Rogosa, and Sharpe agar (MRSA) medium for 24 h, at 27 °C. Then, the cultures were transferred to the 500 mL of De Man, Rogosa, and Sharpe broth (MRSB) medium for an additional 24 h, at 27 °C in order to receive a higher quantity of bacteria biomass. After incubation, the inoculated growth was centrifuged for 15 min at 25 °C (12,000 rpm). The cells were separated from the supernatant and washed 3 times with distilled water by the centrifugation method. The obtained biomass was transferred to the falcon tube and was used in further steps of the experiment.

### 2.3. Experimental Variants

**Figure 1:**
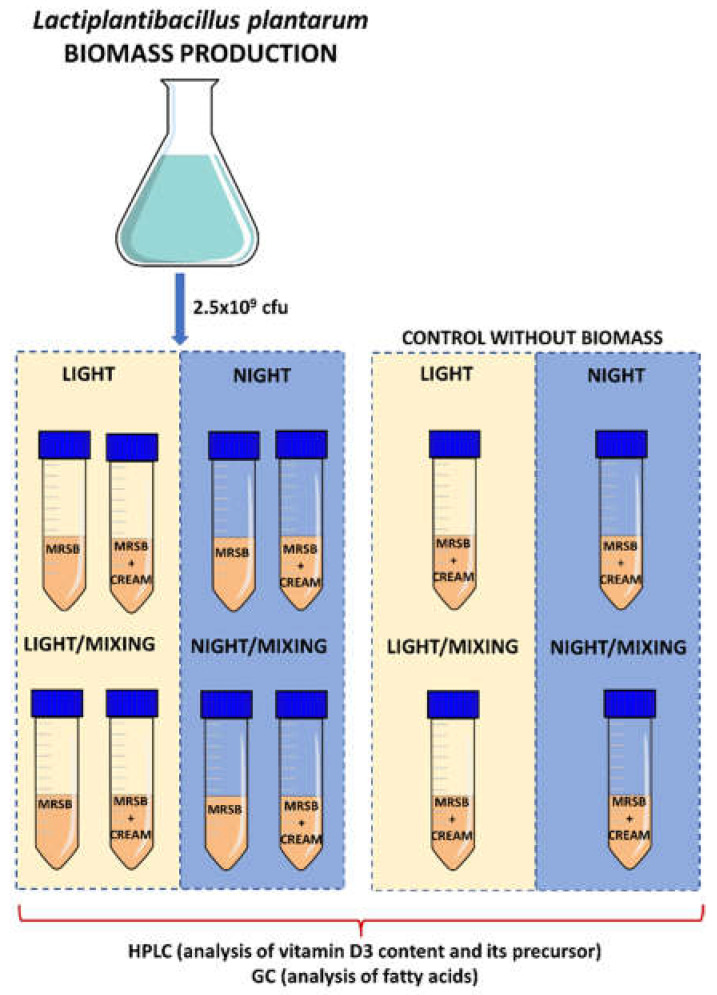
Scheme of the experimental procedure.

The experiment was performed, taking into consideration: (i) biomass addition; (ii) light access, and (iii) mixing. As control samples, cream and MRSB without the addition of *L. paracasei* biomass (LP) were used. As the result, the following variants were investigated: (1) LP in MRSB, night; (2) LP in MRSB, night/mix; (3) LP in MRSB, light; (4) LP in MRSB, light/mix; (5) cream:MRSB—3:1, night; (6) cream:MRSB—3:1, night/mix; (7) cream:MRSB—3:1, light; (8) cream:MRSB—3:1, light/mix; (9) LP in cream:MRSB—3:1, night; (10) LP in cream:MRSB—3:1, night/mix; (11) LP in cream:MRSB—3:1, light; (12) LP in cream:MRSB—3:1, light/mix (Figure 1). Different variants of samples were prepared by adding 2.5 × 10^9^ cfu of *L. paracasei* into the 50 mL falcons containing: 50 mL MRSB (control MRSB), 50 mL cream (control cream), and 37.5 mL cream + 12.5 mL MRSB (cream:MRSB 3:1). Samples were incubated for 48 h at room temperature. The samples in the dark without mixing were incubated in the INCU-Line^®^ Incubator, whereas in the light—on the table under light conditions. Light/mix and night/mix samples were incubated in Julabo SW 22 water bath (150 rpm).All experiments were performed in triplicate.

### 2.4. Sample Preparation and Analysis Procedure for Determination of Fatty Acid Profiles by GC–MS

Extraction of fatty acids was performed using a modified Folch procedure. Briefly, 60 mg of each sample was placed in vials. Then, 2 mL of a chloroform/methanol mixture (2:1, *v*/*v*) was added. In order to increase the extraction efficiency, the samples were ultrasonicated (10 min, at room temperature). Then 0.5 mL of sodium chloride solution (0.05 M NaCl) was added to each sample. The samples were shaken for 10 min on a rotary shaker (200 rpm) and centrifuged for 15 min at 20 °C (2.415× *g*). Finally, the samples were incubated for 15 min to obtain two nonfixed phases. The upper layer was the water fraction and the lower layer was the organic fraction. Both chloroform fractions were collected, pooled, and evaporated to dryness. The dry residue was dissolved in 1 mL of pure methanol. A total of 1 mL of 6% aqueous sodium chloride solution was added to each sample. Samples were vortex mixed for 1 min, then derivatization was performed, as described below. Subsequently, 1 mL of 1% methanolic sulfuric acid solution was added. The chemical conversion process was carried out for 20 min in a water bath at 80 °C. Then the samples were cooled and 1 mL of 6% aqueous sodium chloride solution was added. The fatty acids were extracted in a three-step extraction with hexane. Each step involved adding 1 mL of hexane and mixing on a rotary shaker for 1 min. The extracts were then dried using 1.5 mL of a 4% aqueous potassium carbonate solution. The hexane organic phase was collected and transferred to a new Eppendorf tube and evaporated to dryness. The dry residue was dissolved in 100 µL of methanol and vortexed for 1 min. The samples were centrifuged for 3 min at room temperature (4000 rpm). Then, the samples were introduced to the GC–MS system in the same volume of 1 µL for three replicates.

Analyses were carried out using a GC–mass spectrometry (MS) system from Agilent (6890 A series; Agilent Technologies, Waldbronn, Germany) coupled with a quadrupole mass spectrometer. The derivatized samples were introduced to a gas chromatograph equipped with Agilent (30 m × 0.25 mm i.d., 0.25 µm film thickness) HP-5MS (Agilent) capillary column (Phenomenex, USA). Helium was used as a carrier gas. An injection volume of 5 µL was introduced to the system with the use of split mode (split ratio 50:1) with the ion source temperature held at 230 °C. The oven temperature was set at 50 °C and held for 2 min. Then, the temperature was increased to 230 °C at 5 °C/min and held at a temperature of 230 °C for 8 min to elute any compounds characterized by a high boiling point. The temperature of the MS transfer line was set at 250 °C.

The analyses were performed in the scan mode and MS spectra were recorded in the range of 50–600 *m*/*z*. The obtained data were deconvolved and equalized. Integrated peaks were identified using the NIST 17 library. The percentage content of individual fatty acids was determined in relation to the sum of peak areas of the identified fatty acids in reference to each analyzed sample. In this case, the sum of fatty acids determined in a given sample was 100%. The percentage content of the particular compound identified in a given sample was estimated based on its peak area using a mathematical calculation.

### 2.5. Sample Preparation Procedure and Determination of Vitamin D3 and 7-Dehydrocholesterol Level by HPLC

In the first step, the saponification procedure of the samples was carried out. About 0.15–0.25 g of the sample was weighed into conical flasks. Then 2.5 mL of ethanol (96%) and 0.5 mL of a 60% potassium hydroxide solution were added. The mixture was mixed and placed in a water bath at 80 °C for 15 min. Heating was performed until the solution was completely clear. After the saponification process was completed, the sample was cooled to an ambient temperature and the extraction process was carried out.

The cooled test sample hydrolysate was transferred to a separating funnel and extracted with hexane (5 mL) for 2 min (liquid–liquid extraction). The lower (aqueous) layer was extracted two more times with hexane (2 mL). The pooled hexane extracts were filtered through a filter into which a layer of anhydrous sodium sulfate (about 0.5 g) was applied. The hexane extracts were evaporated to dryness on the Multivap at 36 °C. The dry residue was dissolved in 0.3 mL of ethanol. The solutions prepared in this way were analyzed on HPLC. The vitamin D3 content was read from the standard curve.

Vitamin D3 content was determined by reverse phase HPLC (Shimadzu Prominence) with a DAD detector. In order to prepare a standard curve, 1 mg of vitamin D3 dissolved in 1 mL of ethanol was weighed on an analytical balance. Standard solutions were prepared from the stock solution (1 mg/mL) with a concentration of: 25; 10; 5; 2.5; 1; 0.5; and 0.1 µg/mL. The chromatographic analysis was performed using an ACE 5 C8 column (4.6 × 100 mm), the mobile phase was a mixture of 0.1% formic acid in methanol and 0.1% formic acid in water (90%/10%), the flow rate was 1 mL/min, oven temperature—30 °C, injection volume—5 µL. Vitamin D3 was measured at 265 nm. The time for the vitamin D analysis was 6 min.

Identification of 7-dehydrocholesterol (7-DHC, vitamin D3 precursor) was carried out on Shimadzu HPLC attached to an 8050 QqQ mass spectrometer (Shimadzu) using electrospray ionization (ESI) in a positive ion mode (scan from *m*/*z* 300 to 500). The 7-DHC was separated using a Kinetex C8, with column dimensions of 100 × 2.1 mm, a pore size of 80 Å (Phenomenex, Torrance, CA, USA), a flow rate of 0.3 mL/min, injection volume of 1 µL, separation temperature of 25 °C, and the isocratic mobile phase consisting of 90% methanol and 10% of 0.1% formic acid in water. The ESI settings were as follows: nebulizing gas flow 3 L/min, heating gas flow 10 L/min, the temperature of the drying gas was 400 °C, DL temperature was 250 °C, and interface temperature was 300 °C. The 7-dehydrocholesterol was monitored in the scheduled multiple reaction monitoring (MRM) mode. MRM transitions were 385.5→367.3, collision energy was −19 eV (Q1 = −13 V, Q3 = −14 V).

## 3. Results and Discussion

### 3.1. Impact of the L. paracasei Biomass on the Cream Fatty Acid Composition

**Figure 2:**
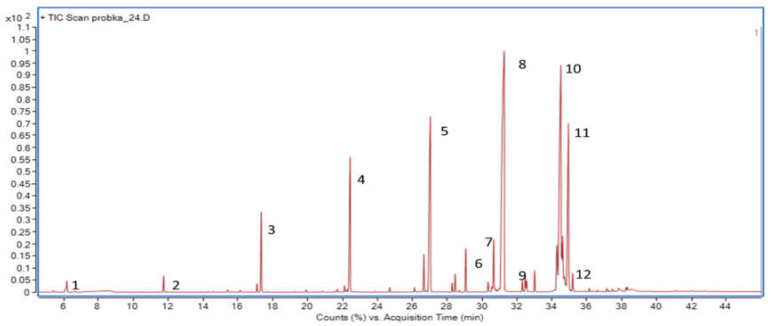
Exemplary chromatogram of fatty acid profiles determined in the biological sample; LP cultured in MRSB:cream—3:1, light. (1) Hexanoic acid; (2) octanoic acid; (3) decanoic acid; (4) dodecanoic acid; (5) tetradecanoic acid; (6) pentadecanoic acid; (7) hexadec-9-enoic acid; (8) hexadecanoic acid; (9) heptadecanoic acid; (10) octadec-9-enoic acid; (11) octadecanoic acid; (12) 9,11-octadecanoic acid.

The results of the FA content analysis revealed the great impact of the investigated *L. paracasei* strain, which varied depending on the applied incubation conditions. An exemplary chromatogram of fatty acid profiles determined in LP cultured in MRSB:cream—3:1 allowed determining hexanoic acid (C6:0), octanoic acid (C8:0), decanoic acid (C10:0), dodecanoic acid (C12:0), tetradecanoic acid (C14:0), pentadecanoic acid (C15:0), hexadec-9-enoic acid (C16:1), hexadecanoic acid (C16:0), heptadecanoic acid (C17:0), octadec-9-enoic acid (C18:1), octadecanoic acid (C18:0), and 9,11-octadecanoic acid (C18:2) (Figure 2).

The results of the FA content analysis revealed the great impact of the investigated *L. paracasei* strain, which varied depending on the applied incubation conditions (Table 1). Regarding MRSB variants, the addition of the *L. paracasei* biomass considerably decreased saturated fatty acid (SFA) content, both in exposure to light and without it (by 26.24 and 17.68%, respectively). Interestingly, the simultaneous use of mixing almost completely eliminated this effect because differences were only up to two percent. Considering unsaturated fatty acids, a different effect was observed depending on the applied light conditions. Under the light exposure, the *L. paracasei* biomass demonstrated the highest impact on monounsaturated fatty acid (MUFA) content (increased by about 22.66%), while under dark conditions, a similar effect was noted for another type of FA—polyunsaturated fatty acids (PUFAs) (increase by ca. 18%). As in the case of SFAs, mixing reduced this effect to a few percent (increase or decrease). Slightly different observations were noted for cream samples. The highest effect of the *L. paracasei* addition was noted when light exposure and mixing were applied together. Such conditions led to a decrease in SFA content (by ca. 7%) and an increase in MUFAs and PUFAs (by 6.15 and 0.95%, respectively). Regarding no light conditions, mixing had the opposite effect: a significant increase of SFAs (~33%) and a simultaneous decrease of MUFAs and PUFAs (31.54 and 1.63%, respectively). In general, the addition of *L. paracasei* caused an increased unsaturated to saturated fatty acid ratio, correlating with the statement that probiotic bacteria could synthesize unsaturated fatty acids upon incubation in milk products [18]. A similar observation was reported by Rodrigues Florence et al. (2009), who observed an increase in PUFAs in milk fermented with *Streptococcus thermophilus* and *Bacillus lactis* [19], as well as by Ewe and Loo (2016), who noted a higher proportion of total unsaturated fatty acids (55%) in butter produced from cream fermented with *L. helveticus* (LH-butter) compared to the control (36%) [14]. The authors pointed out that the milk fat in LH-butter may be hydrolyzed by *L. helveticus* to produce free fatty acids since lactobacilli have been found to possess several intracellular lipases and esterase activities that are responsible for the hydrolysis of fat triglycerides to release free fatty acids.

The GC–MS method enabled the identification of five different MUFAs and four PUFAs in the investigated samples (Table 2). In the experiment, cream mostly contained octadec-9-enoic acid (C18:1) (24.24% of all FA) and hexadec-9-enoic acid (C16:1) (4.41%); nevertheless, three other MUFAs—dodecenoic acid (C12:1), heptadic-10-enoic acid (C17:1), and icos-13-enoic acid (13*c*-20:1) were also detected. Moreover, octadecadienoic acid (C18:2) and octadeca-9,11-dienoic acid (conjugated CLA) were found—1.69 and 0.94%, respectively.

In the MRSB, only octadec-9-enoic acid (4.44%) was noted. After the addition of the *L. paracasei* biomass, the number of different fatty acids increased depending on the incubation conditions used. In the samples kept in the dark, the content of the oleic acid decreased; however, two additional MUFAs appeared—*cis*-9-hexadecenoic acid (C18:1) and 10-heptadecenoic acid (C17:1), as well as elevated amounts of 9,12-octadecadienoic acid (C18:2) (18.08%) in the variant, without mixing. On the contrary, in the light conditions, 10-heptadecenoic acid did not appear, but an amount of the octadec-9-enoic acid increased, particularly in the variant without mixing (six times). Moreover, 9,12-octadecadienoic acid was only detected in the variant with mixing (0.47%).

Considering the impacts of the incubation conditions on the fatty acid compositions of the cream samples, variants without light expositions were characterized by increased octadec-9-enoic acid and paullinic acid content accompanied by a decrease in dodecenoic and 10-heptadecenoic acid levels. Regarding PUFA, the night variant was enriched with 9,12-octadecadienoic acid (almost two times more than the control); as for the night/mix with CLA (~30% more than the control) and eicosa-8,11,14-trienoic acid, as well as arachidonic acid—not detected in the control cream samples. Exposure to light caused a significant decrease in the octadec-9-enoic acid and linoleic acid content in the cream. Moreover, the content of 9-hexadecenoic acid and 10-heptadecenoic acid both decreased and increased depending on whether mixing was applied.

The addition of the *L. paracasei* biomass significantly influenced the distributions of the individual MUFAs in the cream samples. In the case of night and light variants, increased 9-hexadecenoic acid, 10-heptadecenoic acid, as well as octadec-9-enoic acid amounts, were revealed. Contrary to this, mixing decreased both numbers and the amount of MUFAs in the cream samples, except for octadec-9-enoic acid in the light/mixing variant, which significantly increased from 8.87% to 23.15%.

The GC–MS analysis of PUFA content revealed the presence of octadeca-9,12-dienoic acid and conjugated octadeca-9,11-dienoic acid in the cream. Although no PUFAs were identified in the MRSB, the addition of the *L. paracasei* biomass caused octadeca-9,12-dienoic acid to appear in night and light/mixing conditions (18.08 and 0.47%, respectively). Considering cream samples, the highest content of PUFA was noted for night conditions (3.92%). Interestingly, in such conditions, no effect of biomass addition was revealed; in both cases, exactly the same PUFA distribution was noted, 3.16% of octadeca-9,12-dienoic acid and 0.76% of its conjugated form. The greatest diversity of PUFA was noted in the night/mixing variant: CLA, 6,9,12-octadecatrienoic acid, and eicosa-5,8,11,14-tetraenoic acid; however, the total content of PUFA was more than two times lower as in the night conditions. The additions of *L. paracasei* biomass caused the complete disappearance of PUFA in the night/mixing and light conditions. Regarding the light/mixing variant, octadeca-9,12-dienoic acid and conjugated octadeca-9,11-dienoic acid were found; however, their quantities were lower compared to the night variant, 2.39 and 0.71%, respectively. The obtained results indicated that the applied *L. paracasei* strain influenced the fatty acid composition of the cream by changing the MUFA content, mostly causing an increase in octadec-9-enoic acid concentration. Contrary to this, the addition of *L. helveticus* in a study by Ewe and Loo (2016) primarily influenced PUFA content by increasing octadeca-9,12-dienoic acid, while no significant differences regarding MUFA amount were noted. In turn, Karaca et al. (2018) noted higher amounts of docosanoic, heneicosanoic, and eicosadienoic acids after the addition of natural kefir culture into the butter. It suggests that although the effect of adding LAB to the FA compositions of cream and butter is strain-dependent, the addition generally leads to an increase in the unsaturated to saturated fatty acids ratio, which results from the ability of probiotic bacteria to synthesize the unsaturated fatty acids during fermentation [20]. This phenomenon clearly indicates that the addition of LAB into cream increases the beneficial effects of the produced cultured butter on human health by its enrichment with such fatty acids as 9-hexadecenoic acid, octadec-9-enoic acid, octadeca-9,12-dienoic acid, or CLA, which exhibit antimutagenic and anticarcinogenic properties. It is known that octadec-9-enoic acid inhibits the mutagenic activity of food pyrolysate mutagens, polycyclic aromatic hydrocarbons, and nitrosamines [21]. Therefore, the observed increased amount of the octadec-9-enoic acid in cream, with the addition of *L. paracasei*, may play a protective role against the mentioned mutagens via their entrapping within octadec-9-enoic acid micelles. Another beneficial effect of the increased MUFA instead of PUFA content is related to the shelf life of the butter. Although greater polyunsaturated fatty acid content is a major research focus for the dairy industry, the number of double bonds in fatty acids influences the melting behavior and oxidative stability (off-flavors) of the dairy products [22]. Several studies proved that a high content of unsaturated fatty acids in milk fat increases the risk of oxidation and production of off-flavors. This is particularly true of products with high PUFA content that can develop oxidized flavors within 24 h of refrigerated storage [23]. On the contrary, milk fat with a high monounsaturated fatty acid content does not exhibit such oxidation problems [24]. On the other hand, oxidation of milk fat may partially explain the observed generally lower PUFA content in the cream samples incubated under light conditions. So far, there is a lack of studies concerning the effects of the different incubation conditions, such as access to light and mixing on the changes in the FA composition of the fermented cream. As we found, such influence is significant and should not be ignored during the selection of the most suitable LAB strains for cream fermentation and further probiotic butter production. It should be taken into account that light is a source of energy and it is involved in the activation of oxygen that can initiate lipid peroxidation. The fatty acid composition is a very important parameter for samples under light conditions since they are the main substrates for the development of lipid oxidation. Generally, for samples under light conditions and light/mixing conditions, the levels of SFA were lowered while the MUFA levels increased. This is a favorable phenomenon because MUFAs are almost completely insensitive to peroxidation. A study by Lin et al. [25] revealed that some LAB, especially *L. plantarum*, showed strong antioxidant activity; this was revealed by a high scavenging effect on hydrogen radicals and inhibition of lipid peroxidation; in this case, the mechanism has not yet been clarified.

### 3.2. Impact of the L. paracasei Biomass on Vitamin D3 and Its Precursor Content in Cream

As a result of the validation of the method used for the determination of vitamin D3 content, a calibration curve was obtained, characterized by linearity in the range of 0.5–25 µg/mL (R^2^ = 0.999, LOD = 0.1 µg/mL, LOQ = 0.33 µg/mL) that enabled measuring the vitamin D3 content in the investigated samples.

**Figure 3:**
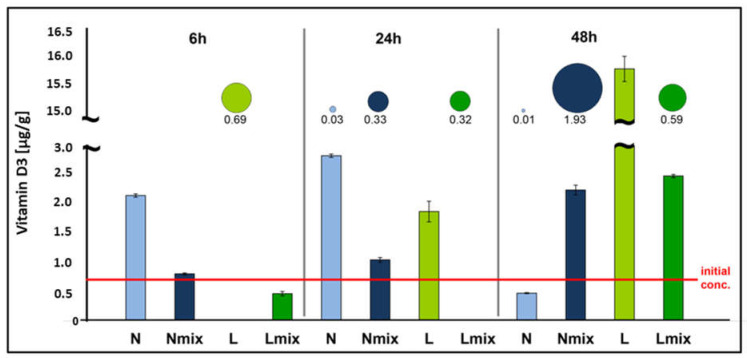
Effects of the addition of *L. paracasei* strain Parakas fresh biomass on the changes in the vitamin D3 content (bars) and its precursor (bubbles) in cream under different incubation conditions. N—night; Nmix—night/mixing; L—light; Lmix—light/mixing. The diameters of the bubbles are proportional to the concentration of the precursor (µg/g).

The initial amount of vitamin D3 in the cream was regarded as 0.8 µg/g, which was measured at the starting point of the experiment. An analysis revealed a significant impact of the *L. paracasei* biomass addition on the changes in the vitamin D3 content in the cream samples (Figure 3). Such an effect strongly depended on the applied incubation conditions and time. Regarding the variants without access to light, mixing caused a gradual increase in vitamin content starting from 24 h of incubation reaching a value of 2.2 µg/g. In variants without mixing, vitamin D3 content after 6 and 24 h was 2.6–3.5 higher compared to the control, respectively; at the end of the experiment, it decreased below the initial amount, reaching a value a 0.5 µg/g. In both light variants, vitamin D3 content decreased initially after 6 h of incubation and then increased significantly after 24 h (light variant) and 48 h (both with and without mixing). The highest increase was noted for conditions with light and without mixing; between 24 and 48 h of incubation of vitamin D3 content elevated 8.3 times (from 1.9 to 15.7 µg/g).

Regarding the precursor, the obtained calibration curve was characterized by linearity in the range 0.25–25 µg/mL (R^2^ = 0.997, LOD = 0.05 µg/mL, LOQ = 0.165 µg/mL). An analysis revealed great variation in the precursor concentration in the cream depending on the experimental variant and time (bubbles in Figure 3). The greatest variation was observed between mixing and no mixing conditions, where in mixed variants, the precursor content increased during incubation time while the opposite phenomenon for samples without agitation was noted. For mixed samples kept in the dark, the precursor content increased from 0 µg/g (6 h) to 1.93 µg/g (48 h) and was strongly positively correlated with the vitamin D3 content (r = 0.999, *p* = 0.003). A similar phenomenon was noted for the light/mixing variant where the precursor concentration increased from 0 to 0.59 µg/g and was positively correlated with the vitamin D3 content (r = 0.724); however, such a correlation was not statistically significant (*p* = 0.485). Regarding no mixing variants, the presence of the precursor under light conditions was observed only after 6 h of incubation (C = 0.69 µg/g), while in the night variants, only small amounts (after 24 and 48 h) were measured—0.03 and 0.01 µg/g, respectively.

**Figure 4:**
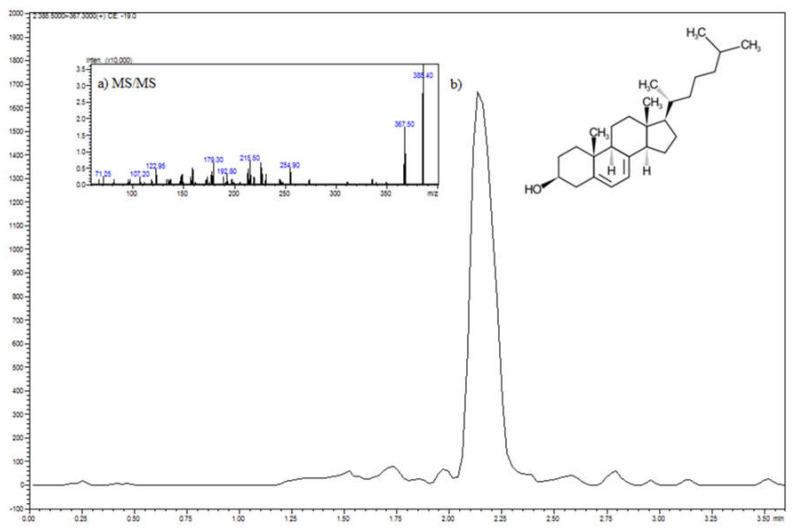
Mass spectra (**a**) and chromatogram (**b**) of 7−dehydrocholesterol.

It is commonly known that vitamin D3 biosynthesis starts from the oxidation of the cholesterol molecules into the 7-DHC, called provitamin D3 (Figure 4). Subsequently, 7-DHC undergoes photolytic conversion to cholecalciferol (pre-vitamin D3), which almost instantly isomerizes at room temperature to vitamin D3. During this process, the key factor is the absorption of UVB radiation (290–315 nm), which breaks the double bond at carbons 9 and 10 and, consequently, opens the B ring [26]. Since cream contains substantial amounts of cholesterol [27], such a phenomenon may explain the observed highest content of vitamin D3 in light conditions, which is also related to the biocolloidal nature of milk fat. The disproportion in vitamin D concentrations under light conditions, depending on the static and dynamic process (light/mix) and the presence of bacteria cells, most likely result from the local concentrations in milk fat micelles and the dynamics of the system dispersion. The properties of biocolloids are often used in the formulation of lipid nanocapsules for milk fortification characterized by a high level of dispersion homogeneity [28]. To avoid the heterogeneity of dispersion in dairy products, the high-pressure treatment on the dispersion of fat micelles was applied [29]. Therefore, the highest content of vitamin D in light conditions is also associated with the chemistry of milk fats. Light induces the formation of free radicals in the cream system, which cause lipid peroxidation (LPO) [30]. It is a free radical oxidation process of unsaturated fatty acids [31]. The contribution of this phenomenon is evidenced by the reduction of PUFA concentration and an increase in the content of saturated fatty acids for the variant with the highest concentration of vitamin D.

The initiation of lipid peroxidation consists of the detachment of the hydrogen atom from the polyunsaturated fatty acid molecule or the rest of this acid that is part of the cell wall of the tested LAB. The research by Xiangna Lin et al. [25] demonstrated antiradical properties and the lipid peroxidation inhibiting effect of the *Lactobacillus plantarum* strain. Unsaturated fatty acids in cell membranes are easily attacked by free radicals. Lipid peroxidation can be initiated by a hydroxyl radical (OH•), and radicals: peroxide (LO•), alkoxy (LO•), or alkyl (L•) [31]. During the propagation reaction, free radicals react with oxygen and form LOO• free peroxide radicals. These, in turn, can detach hydrogen atoms from subsequent molecules of LH polyunsaturated fatty acids. In this reaction, the free radical does not die but reacts with all species present in the studied colloidal system: fat particles, bacterial cell walls, and the vitamin D precursor. As a result of the interaction formed, the photolytic conversion of 7-DHC to cholecalciferol, whereupon 7-DHC is isomerized almost immediately at room temperature to form vitamin D3.

**Figure 5:**
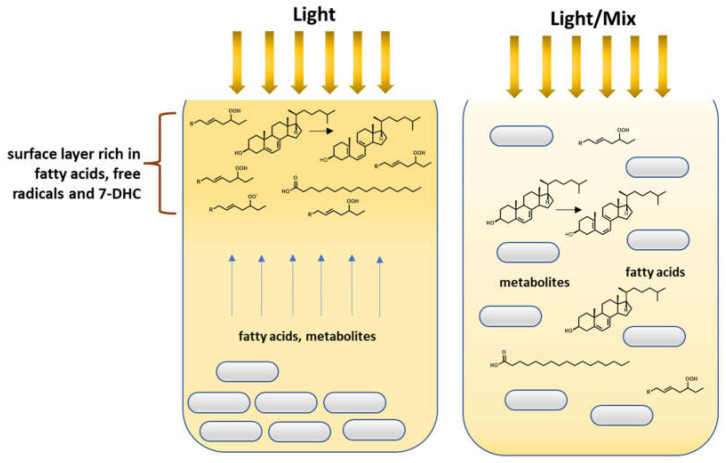
The scheme presents the hypothesis of various components of cream distribution during the conversion of 7-dehydrocholesterol to vitamin D3.

Mixing has a key impact on the processes taking place with the participation of microorganisms on an industrial scale. Generally, the faster growth of cells in the samples subjected to mixing shows more favorable conditions because of easier and uniform access to carbon sources and other ingredients necessary for the proper growth and development of cells. However, in our study, during vitamin D3 synthesis, an inverse relationship was observed [32,33]. Mixing had a significant role in the processes modulated by the *L. paracasei* strain as it significantly limited the contribution of light. The explanation could be that during a stationary process (without mixing), fatty acids and vitamin D3 precursors that accumulate on the cream’s surface layer are exposed longer to light than during mixing, where they are evenly distributed throughout the volume (Figure 5).

## 4. Conclusions

The results showed that the addition of the *L. paracasei* strain Parakas to cream modulates the profile of fatty acids as well as the level of provitamin D3 and vitamin D3. The obtained profiles of fatty acids and the amounts of provitamin D3 and vitamin D3 strongly depended on the presence of light and the mixing of the sample during incubation with *L. paracasei*. These factors through changes in the level of free radicals can affect the metabolism of bacteria. In general, the presence of *L. paracasei* contributed to an increased unsaturated to saturated fatty acids ratio, agreeing with the statement that probiotic bacteria could synthesize unsaturated fatty acids upon incubation in milk products. It is worth mentioning that this LAB enriched the cream in 9-hexadecenoic acid, octadec-9-enoic acid, octadeca-9,12-dienoic acid, and CLA. A higher level of MUFA in the cream can, in the future, extend the shelf life of butter and, at the same time, improve its health-promoting properties. The presence of LAB after 6 h contributed to an increase in the level of provitamin D, and an increase in the level of vitamin D3 after 24 and 48 h. The light was probably responsible for lipid peroxidation processes occurring in the butter, which could modulate the interaction of *L. paracasei* with the ingredients of the cream.

## Figures and Tables

**Table 1:** Effect of the addition of the *L. paracasei* strain Parakas biomass on the changes in the fatty acid content (SFA—saturated fatty acid; MUFA—monounsaturated fatty acid; PUFA—polyunsaturated fatty acid), under different incubation conditions. MRSB—De Man, Rogosa, and Sharpe Broth; LP—*Lacticaseibacillus paracasei* strain Parakas.

			Fatty Acids [%]			
Variant	SFA	Regulation vs. Control	MUFA	Regulation vs. Control	PUFA	Regulation vs. Control
Cream	66.84		30.53		2.63	
MRSB	95.56		4.44		0.00	
LP in MRSB, night	77.87	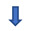	4.04	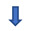	18.08	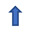
LP in MRSB, night/mix	96.20	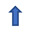	3.80	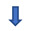	0.00	
LP in MRSB, light	69.32	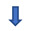	27.10	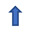	3.58	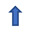
LP in MRSB, light/mix	93.42	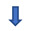	6.10	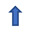	0.47	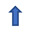
Cream:MRSB—3:1, night	68.74		27.82		3.44	
Cream:MRSB—3:1, night/mix	62.38		35.98		1.63	
Cream:MRSB—3:1, light	80.38		19.20		0.43	
Cream:MRSB—3:1, light/mix	78.02		19.83		2.15	
LP in Cream:MRSB—3:1, night	65.92	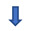	30.17	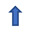	3.92	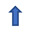
LP in Cream:MRSB—3:1, night/mix	94.10	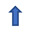	5.90	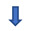	0.00	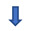
LP in Cream:MRSB—3:1, light	77.03	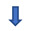	22.97	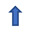	0.00	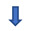
LP in Cream:MRSB—3:1, light/mix	70.92	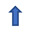	25.98	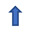	3.10	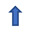
		Conditions with the highest impact on the FA content				
	SFA ↓		MUFA ↑		PUFA ↑	
LP in MRSB	light		light		night	
LP in Cream:MRSB	light/mix		light/mix		light/mix	

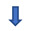
 decrease in fatty acid content compared to control samples (MRSB and Cream:MRSB—3:1); 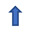 increase in fatty acid content compared to control samples (MRSB and Cream:MRSB—3:1).

**Table 2:** The content of individual monounsaturated fatty acids (MUFAs) and polyunsaturated fatty acids (PUFAs) are expressed as percentages of all fatty acids in the sample, depending on the light/mixing conditions used as well as *L. paracasei* strain Parakas biomass addition. **—Statistically significant differences (*p* ≤ 0.001) between the inoculated and corresponding uninoculated variants according to one-way ANOVA and the Newman–Keuls post hoc test. C12:1—dodecenoic acid; C16:1—hexadec-9-enoic acid; C17:1-heptadec-10-enoic acid; C18:1—octadecenoic acid; C20:1—icos-13-enoic acid; C18:2—octadeca-9,12-dienoic acid; C20:2—octadeca-9,11-dienoic acid (conjugated linoleic acid, CLA); C20:3—eicosa-8,11,14-trienoic acid; C20:4—eicosa-5,8,11,14-tetraenoic acid.

	Variant					MUFA [%]										PUFA [%]			
		C12:1	RSD	C16:1	RSD	C17:1	RSD	C18:1	RSD	C20:1	RSD	C18:2	RSD	C20:2	RSD	C20:3	RSD	C20:4	RSD
	Cream	0.74 ± 0.015	2.02	4.41 ± 0.0009	0.02	1.15 ± 0.019	1.65	24.24 ± 0.36	1.49	0.25 ± 0.008	3.2	1.69 ± 0.055	3.25	0.94 ± 0.016	1.7	-		-	
	MRSB	-		-		-		4.44 ± 0.25	5.6	-		-		-		-		-	
LP in MRSB	night	-		0.31 ± 0.01	3.23	0.79 ± 0.019 **	2.4	2.95 ± 0.17	5.76	-		18.08 ± 0.66 **	3.65	-		-		-	
	night/mix	-		-		-		3.8 ± 0.21	5.52	-		-		-		-		-	
	light	-		7.91 ± 0.19 **	2.4	-		19.2 ± 0.28 **	1.45	-		-		-		-		-	
	light/mix	-		0.26 ± 0.02	7.69	-		5.84 ± 0.12	2.05	-		0.47 ± 0.009	1.92	-		-		-	
Cream	night	-		1.99 ± 0.018	0.9	0.39 ± 0.011	2.82	25.15 ± 0.18	0.72	0.3 ± 0.006	2.00	3.16 ± 0.022	0.70	0.76 ± 0.018	2.37	-		-	
	night/mix	0.36 ± 0.013	3.61	4.85 ± 0.023	0.47	1.02 ± 0.017	1.66	29.76 ± 0.28	0.94	0.67 ± 0.009	1.34	-		1.25 ± 0.019	1.52	0.21 ± 0.007	3.33	0.18 ± 0.011	6.11
	light	0.39 ± 0.009	2.31	1.61 ± 0.015	0.93	0.27 ± 0.005	1.85	16.93 ± 0.014	0.08	-		0.43 ± 0.008	1.86	-		-		-	
	light/mix	0.72 ± 0.012	1.66	8.78 ± 0.17	1.94	1.46 ± 0.019	1.30	8.87 ± 0.16	1.80	1.02 ± 0.016	1.57	-		2.15 ± 0.025	1.16	-		-	
LP in Cream	night	0.35 ± 0.01 **	2.86	2.57 ± 0.008 **	0.3	0.49 ± 0.006 **	1.22	26.75 ± 0.15 **	0.56	- **		3.16 ± 0.21	6.65	0.76 ± 0.013 - **	17.11			-	
	night/mix	- **		- **		- **		5.90 ± 0.09 **	1.53	- **		-		-		- **		- **	
	light	- **		1.88 ± 0.19 **	1.00	0.37 ± 0.008 **	2.16	20.72 ± 0.15 **	0.72	-		- **		-		-		-	
	light/mix	- **		2.41 ± 0.25 **	10.37	0.42 ± 0.009 **	2.14	23.15 ± 0.21 **	0.91	- **		2.39 ± 0.10 **	4.18	0.71 ± 0.016 **	2.25	-		-	

## Data Availability

Data is contained within the article.
